# Breast Reduction Mammaplasty

**Published:** 2013-09-19

**Authors:** Karan Chopra, Kashyap K. Tadisina, Devinder P. Singh

**Affiliations:** ^a^Department of Plastic and Reconstructive Surgery, The Johns Hopkins Hospital, Baltimore, Md; ^b^University of Illinois at Chicago College of Medicine, Chicago, Ill; ^c^Division of Plastic Surgery, University of Maryland School of Medicine, Baltimore, Md

**Keywords:** gigantomastia, inferior pedicle reduction mammaplasty, large volume breast reduction, macromastia, wise pattern mammaplasty

**Figure:**
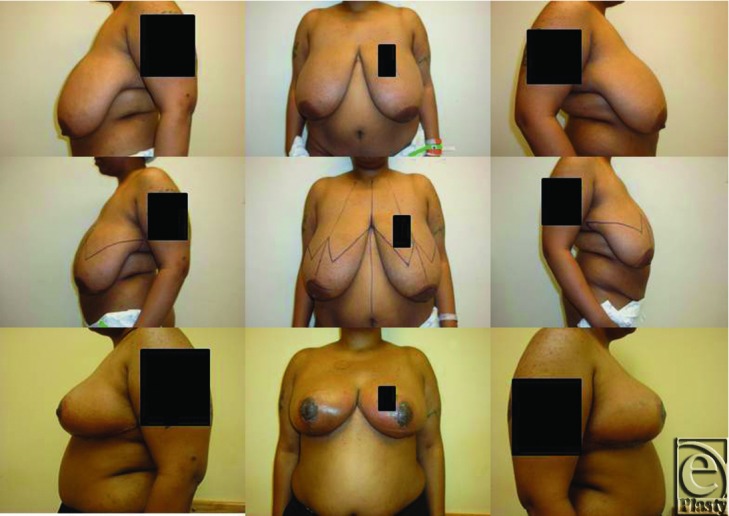


## DESCRIPTION

A 45-year-old obese woman presented for evaluation of her symptomatic large breasts. After consultation, the patient elected to undergo bilateral Wise pattern breast reduction with inferior pedicle.

## QUESTIONS

1. **When is breast reduction indicated and what are the most popular techniques used?**
2. **What are advantages/disadvantages of the Wise pattern inferior pedicle technique?**
3. **What are the advantages and disadvantages of liposuction reduction?**
4. **What are common complications of breast reduction?**

## DISCUSSION

Reduction mammaplasty is one of the most common procedures performed by plastic surgeons today.^1^ Reduction is indicated for patients with symptomatic breast hypertrophy. Large ptotic breasts can quickly become troublesome for patients, leading to potentially debilitating symptoms and a poor quality of life. Patients seeking breast reduction commonly present with complaints of back pain, neck pain, severed discomfort, unwanted harassment, and poor self-image, among other symptoms. These patients can benefit greatly from a reduction in breast size, as most symptoms are relieved by reduction mammaplasty.^2^^,^^3^ The inferior pedicle-based Wise pattern reduction mammaplasty technique is the most popular reduction method used by plastic surgeons in the United States. According to a 2006 survey of plastic surgeons, 69% used the Wise pattern reduction technique as their preferred mammaplasty technique. Besides the Wise pattern technique, the short scar reduction technique is the most popular option, especially in European countries. However, due to several limitations, including a steeper learning curve, inability to visualize the final outcome in the early postoperative period, high re-operation rate, and increased risk of litigation, it is less popular in the United States.^4^

The Wise pattern technique offers surgeons versatility and wide access to the breast parenchyma with the inverted T incision, allowing this technique to be used effectively for a large variety of reduction sizes, especially high-volume reductions. In addition to its versatility, this technique also yields predictable and reliable results, including intact nipple sensitivity. Furthermore, patients undergoing reduction with Wise pattern mammaplasty report satisfaction rates comparable to those of other techniques.^5^^,^^6^ One of the drawbacks to the Wise pattern technique, however, is that compared to a smaller incision technique, there is a more obvious scar, some of which can become hypertrophic. Furthermore, Wise pattern breast reductions are more prone to wound complications at the inverted T closure and have a tendency to start “bottoming out” postoperatively, or become wider and flat, with nipples pointing superiorly, leaving the superior pole with an “empty” deflated appearance.^5^^,^^6^

For women who are suffering from symptoms that are based mainly on breast weight and less so by ptosis, liposuction presents them with a viable and unique option. Liposuction is minimally invasive compared to reduction mammaplasty surgical procedures and results in relatively smaller scars, as well as a chance for skin to retract naturally. Older women are better candidates for liposuction techniques, as they have a larger percentage of fatty tissue. However, the scope of this procedure is limited to a very small subset of patients, as it does not allow for glandular tissue removal, and has a steep learning curve. Some controversy also surrounds the process of adding or taking adipose tissue out of a female breast, as increased oncologic risk has not been disproved.^7^

As with any surgical procedure, there are potential complications associated with breast reduction. These complications include asymmetry, dissatisfaction with reduction amount, or disappointment with shape and projection. Other complications that can result are wound dehiscence, infection or cellulitis, necrosis, hypertrophic scarring, loss of nipple sensitivity, reduced ability to breast feed, or complete loss of nipple-areolar tissue.^8^^,^^9^ With the rapid growth of the obesity epidemic, the risk of complications such as dehiscence and infection are more likely to increase.

Breast reduction mammaplasty is one of the most common procedures performed by plastic surgeons in the United States. Despite many surgical options, most plastic surgeons use the reliable and easy to learn Wise pattern technique. However, other techniques such as liposuction reduction are also available to select patient populations. Given the rise of obesity in the American population, complications related to breast reduction in cases of severe macromastia will continue to be a concern for plastic surgeons. This prevalence of massive gigantomastia has led to innovations in reduction mammaplasty and the implementation of newer techniques such as modifications to the traditional Wise pattern technique and the use of intraoperative laser angiography techniques to assess nipple viability.
